# Immunohistochemical analysis of SHH, SMO and GLI-1 proteins in epithelial odontogenic lesions

**DOI:** 10.1590/0103-6440202204972

**Published:** 2022-10-21

**Authors:** Katianne Soares Rodrigues, Hellen Bandeira de Pontes Santos, Everton Freitas de Morais, Roseana de Almeida Freitas

**Affiliations:** 1 Department of Oral Pathology, Federal University of Rio Grande do Norte, Natal, Rio Grande do Norte, Brazil

**Keywords:** Odontogenic cysts, Odontogenic tumors, Hedgehog proteins, Immunohistochemistry

## Abstract

The present study analyzed the expression of proteins involved in the sonic hedgehog signaling pathway (SHH, SMO, and GLI-1) in benign epithelial odontogenic lesions (odontogenic keratocyst - OKC, ameloblastoma - AB, and adenomatoid odontogenic tumor - AOT) in order to identify the role of these proteins in the pathogenesis of these lesions. The sample consisted of 20 OKCs, 20 ABs, and 10 AOTs. The Kruskal-Wallis, Mann-Whitney *U*, and Spearman’s (*r*) tests were used for statistical analysis, with the level of significance set at 5% (*p* < 0.05). The membrane/cytoplasmic expression of SHH was significantly higher in AB compared to AOT (*p* = 0.022) and OKC (*p* = 0.02). No differences were found in the membrane/cytoplasmic expression of SMO between the lesions studied. Regarding GLI-1, significant differences were observed at the nuclear level for AB and OKC compared to AOT (*p* < 0.0001). In addition, significant positive correlations were found between cytoplasmic and nuclear GLI-1 in AB (*r* = 0.482; *p* = 0.031) and OKC (*r* = 0.865; *p* < 0.0001), and between membrane/cytoplasmic SMO and cytoplasmic GLI-1 in AOT (*r* = 0.667; *p* = 0.035) and OKC (*r* = 0.535; *p* = 0.015). The results of this study confirm the participation of the sonic hedgehog signaling pathway in the pathogenesis of the lesions studied. Overexpression of SHH in ABs and nuclear expression of GLI-1 in ABs and OKCs indicate that these proteins contribute to the more aggressive behavior of these two lesions when compared to AOT.

## Resumo

O presente estudo analisou a expressão de proteínas envolvidas na via de sinalização Sonic Hedgehog (SHH, SMO e GLI-1) em lesões benignas do epitélio odontogênico de comportamento biológico distintos, tais como ceratocistos odontogênicos (CO), ameloblastomas (AMB) e tumores odontogênicos adenomatoides (TOA), com o intuito de identificar o papel destas proteínas na patogênese destas lesões. A amostra foi constituída de 20 CO, 20 AMB e 10 TOA, analisada pela técnica da imuno-histoquímica de forma semiquantitativa por compartimento celular, onde foi feita uma análise da membrana e citoplasma das células nas proteínas SHH e SMO, enquanto que para a proteína GLI-1, foi feita uma análise nuclear e/ou citoplasmática. Para análise estatística, foram utilizados os testes de Kruskal-Wallis (KW), Mann-Whitney (U) e Spearman (r), com o nível de significância estabelecido em 5% (p < 0,05). Ao analisar a proteína SHH, observou-se que o AMB demonstrou expressão membranar/citoplasmática significativamente maior em comparação ao TOA (p = 0,022) e CO (p = 0,020). Com relação à análise membranar/citoplasmática da SMO, não foram identificadas diferenças entre as lesões estudadas. Para a proteína GLI-1, foram constatadas diferenças estatisticamente significativas, em nível nuclear, para o AMB e CO em comparação ao TOA (p< 0,0001). Além disso, foram observadas correlações positivas com significância estatística entre GLI-1 citoplasmático e GLI-1 nuclear para o AMB (r = 0,482; p = 0,031) e CO (r = 0,865; p< 0,0001), e entre o SMO membranar/citoplasmático e o GLI-1 citoplasmático para o TOA (r = 0,667; p = 0,035) e o CO (r = 0,535; p = 0,015). Os resultados deste estudo confirmam a participação da via de sinalização Sonic Hedgehog na patogênese das lesões estudadas e, a superexpressão de SHH em AMBs e GLI-1 nuclear em AMBs e COs, indica que estas proteínas contribuem com o comportamento biológico mais agressivo destas duas lesões quando comparado ao TOA.

## Introduction

Tooth development comprises different stages of morphodifferentiation and histodifferentiation in an event called odontogenesis. This event is controlled by a series of sequential signaling interactions between the epithelial lining of the primitive oral cavity and the ectomesenchyme derived from the neural crest. Epithelial remnants of odontogenesis can be found trapped inside the jaw bones or in adjacent tissues at the end of this process. These remnants have the potential to cause lesions such as odontogenic cysts and tumors ^1^ ^,^ ^2^ ^,^ ^3^ ^,^ ^4^.

Among developmental odontogenic cysts, the odontogenic keratocyst (OKC) deserves attention because of its aggressive biological behavior, high recurrence rates, specific histopathological features, and its association with nevoid basal cell carcinoma in some cases ^5^ ^,^ ^6^ ^,^ ^7^.

Regarding odontogenic tumors, ameloblastoma (AB) is particularly important because of its high prevalence. It is the most common lesion in the group of tumors derived from the odontogenic epithelium. In addition, this tumor has been the subject of ongoing investigations because of its locally aggressive behavior and tendency to recur. In contrast to AB, adenomatoid odontogenic tumor (AOT) is an epithelial odontogenic tumor characterized by slow and progressive growth and an indolent biological and clinical behavior, with no tendency to recur ^7^ ^,^ ^8^ ^,^ ^9^ ^,^ ^10^.

Controversy exists in the literature regarding the pathogenesis of odontogenic lesions. Thus, many studies have tried to identify and demonstrate the role of the molecular events underlying the development and progression of these lesions. For example, genetic and epigenetic studies have investigated different signaling pathways, particularly the sonic hedgehog (Shh) pathway ^1^ ^,^ ^11^ ^,^ ^12^ ^,^ ^13^ ^,^ ^14^. The Shh signaling pathway has been extensively studied in odontogenic cysts and tumors because it plays a key role in the embryonic development of the tooth. In addition, the relationship between mutations in some genes involved in this pathway, such as PTCH1 and SMO in OKCs and ABs, is well established in the literature, indicating the participation of these genes in the development of these lesions ^2^ ^,^ ^5^ ^,^ ^13^ ^,^ ^15^.

In view of the above considerations and the continued studies that may contribute to a better understanding of the pathogenesis of these odontogenic lesions, this study investigated the immunoexpression of Shh, SMO and GLI-1 proteins in odontogenic lesions with distinct biological behaviors, including OKC, AB and AOT.

## Materials and Methods

### Ethical Considerations

The study was approved by the Ethics Committee of the Federal University of Rio Grande do Norte (UFRN) through Plataforma Brasil, and was conducted in accordance with Resolution 466/12 of the National Health Council (number 387.912).

### Sample

The sample consisted of 50 cases of odontogenic lesions, including 20 cases with a histological diagnosis of non-syndromic OKC, 20 cases of conventional AB, and 10 cases of AOT. The cases seen between 1980 and 2019 were retrieved from the archives of the Laboratory of Pathological Anatomy and Cytopathology, Discipline of Oral Pathology, Department of Dentistry, UFRN. All cases selected were revised by oral pathologists and exhibited well-preserved histopathological features and sufficient biological material for immunohistochemistry. Cases showing an intense inflammatory component, which can cause distortions in the epithelial component of the lesions, were excluded. Clinical data (sex, age, and anatomical site) were collected from the biopsy records stored at the service and are shown in Table 1.

**Table 1:** Frequency of cases of ABs, AOTs and OKCs according to gender, age and anatomical location.

Injury/Variable	Total n (%)
Ameloblastoma	
*Sex*	
Female	11 (55.0)
Male	9 (45.0)
*Age*	
Average (±SD)	30.05 (±21.00)
*Location*	
Mandible	16 (80.0)
Maxilla	4 (20.0)
	
**Adenomatoid Odontogenic Tumor**	
*Sex*	
Female	6 (60.0)
Male	4 (40.0)
*Age*	
Average (±SD)	16.80 (±12.99)
*Location*	
Mandible	3 (30.0)
Maxilla	7 (70.0)
	
Odontogenic keratocysts	
*Sex*	
Female	8 (40.0)
Male	12 (60.0)
*Age*	
Average (±SD)	32.10 (±12.52)
*Location*	
Mandible	17 (85.0)
Maxilla	3 (15.0)

Abbreviations: OKCs, Odontogenic keratocysts; AOTs, Adenomatoid Odontogenic Tumors; ABs, Ameloblastomas; SD, Standard deviation.

### Immunohistochemical Analysis

The tumor specimens fixed in 10% formalin and embedded in paraffin were cut into 3-µm sections. The sections were mounted on clean and defatted glass slides prepared with organosilane adhesive (3-aminopropyltriethoxy-silane, Sigma Chemical Co., St. Louis, MO, USA). For deparaffinization and antigen retrieval, the histological sections were pretreated with Trilogy solution (1:100; Cell Marque, Rocklin, CA, USA) in an electric pressure cooker for 3 min. The slides were incubated two times (15 min each) in hydrogen peroxide solution to block endogenous peroxidase, followed by incubation with Protein Block (Dako, Carpinteria, CA, USA) for 5 min. Next, the sections were washed twice in Tris-Tween 20, pH 7.4, for 5 min each.

Each case was incubated with the following primary antibodies overnight (18 hours): SHH (EP1190Y, Abcam Discover More; 1:2,500), SMO (ab72130, Abcam Discover More; 1:2,500), and GLI-1 (EPR4523, Abcam Discover More; 1:500). Next, the sections were treated with the HiDef Detection HRP Polymer System (Cell Marque, Rocklin, CA, USA) for 20 min. After several washes, diaminobenzidine (Liquid DAB + Substrate, Dako) was applied as chromogen for 5 min. The sections were counterstained with Mayer’s hematoxylin (Dako).

Immunohistochemical analysis was performed by a previously trained examiner at two different time points. Only the tumor parenchyma (AB and AOT) and cystic epithelial lining of OKC were analyzed. The criteria established for analysis were localization in the cellular compartments and negative and positive staining. For this purpose, cells exhibiting brown staining irrespective of intensity were classified as positive. Immunoreactivity was evaluated semiquantitatively according to cellular compartment as described by Vered et al, where 0 = absence of staining, 1 = 0 to 10% of positive cells (low), 2 = 11 to 50% of positive cells (intermediate), and 3 ≥ 50% of positive cells (high). Regarding cellular compartment, SHH and SMO were analyzed in the membrane and cytoplasm, while GLI-1 was analyzed in the nucleus and/or cytoplasm.

### Statistical Analysis

The results were compiled in a database organized in Microsoft Excel® spreadsheets and analyzed with the SPSS® for Windows 20.0 software (Statistical Package for the Social Sciences, Inc., Chicago, USA). Descriptive statistics was used for characterization of the sample. The immunopositivity scores were analyzed by the Kolmogorov-Smirnov test, which revealed the absence of a normal distribution. Thus, the nonparametric Kruskal-Wallis (KW) and Mann-Whitney (*U*) tests were used for comparison of the immunoexpression scores for SHH, SMO and GLI-1 between the lesions studied. Correlations between the expression of the proteins were analyzed using Spearman’s correlation (*r*) test. The level of significance was set at 5% (*p* < 0.05) in all statistical tests.

## Results

The present sample consisted of 20 OKCs, 20 ABs, and 10 AOTs. There was a predominance of female cases with AB and AOT, while OKCs were predominant in males. The mean age was 32 years (range 12 to 63 years) in patients with OKC, 30.05 years (range 13 to 69 years) in patients with AB, and 16.8 years (range 13 to 51 years) in patients with AOT. Regarding anatomical location, the posterior region of the mandibular body and ramus was the most affected site in cases of OKC and AB, while AOTs more frequently involved the anterior maxilla. Table 1 shows the clinical and epidemiological profile of each lesion.

### Immunoexpression profile of SHH, SMO and GLI-1

**Figure 1:**
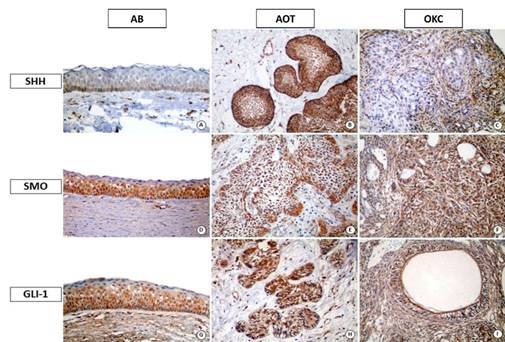
Immunoexpression of SHH, SMO and GLI-1 proteins in odontogenic keratocyst (OKC), ameloblastoma (AB), and adenomatoid odontogenic tumor (AOT) (magnification 400×). (A) Membrane/cytoplasmic expression only in the basal layer of the epithelial lining of OKC. (B) high membrane/cytoplasmic expression in AB. (C) Intermediate staining of the protein only at the membrane/cytoplasmic level in AOT. (D) high cytoplasmic/membrane expression, except for the superficial cell layer of the epithelium in OKC. (E) Intense cytoplasmic/membrane immunostaining in AB. (F) high staining only in the membrane/cytoplasmic compartments of AOT. (G) high nuclear expression, except for the superficial cell layer of the cystic epithelium in OKC. (H) Nuclear immunostaining in nests of the odontogenic epithelium in AB. (I) high and indistinct immunohistochemical expression only in the cytoplasm of AOT.

Regarding the expression of SHH in OKC, immunoreactive cells were mainly located in the basal layer, with the observation of low or high immunoexpression. In ABs, SHH was highly expressed in most cases. In some cases of the follicular and plexiform patterns, immunostaining was stronger in columnar or cuboidal peripheral cells than in loosely arranged central cells. Most AOTs analyzed exhibited low or absent staining. When positive, tumor cell staining was found to be indiscriminate. Membrane/cytoplasmic staining of SHH was observed in the three lesions studied (Figure 1).

Analysis of SMO revealed high immunohistochemical expression in all OKC cases, with the observation of brown staining in cells of all layers of the epithelial lining of this cyst, except for the superficial parakeratin layer. In cases of AB, stronger staining was detected in columnar or cuboidal peripheral cells when compared to the loosely arranged central cells in the follicular and plexiform patterns. However, indistinct staining was observed in some cases. The AOT cases studied exhibited high and indistinct staining of tumor cells. Analysis according to cellular compartment showed membrane/cytoplasmic staining in all cases of the three lesions (Figure 1).

Finally, specifically regarding the expression of GLI-1, OKCs exhibited high immunohistochemical expression in most cases. All layers of the cystic epithelial lining, except for the superficial parakeratin layer, were immunostained. It is also important to point out that basal layer cells were intensely stained in all cases. In the group of ABs, indistinct and high immunohistochemical expression of GLI-1 was observed in all cases of the follicular and plexiform patterns. All AOT cases exhibited high and indiscriminate immunoexpression in tumor cells. Regarding location, nuclear and/or cytoplasmic expression was observed in ABs and OKCs, while all cases of AOT only exhibited cytoplasmic immunoreactivity (Figure 1).

Membrane/cytoplasmic immunoexpression of SHH was observed in all AB and OKC cases (100%) and in seven AOT cases (70%). The nonparametric KW test revealed a significant difference in the median immunoexpression scores between the groups of lesions (*p* ≤ 0.011) (Table 2). The Mann-Whitney test was applied to identify where this difference occurred (Table 3), which revealed significantly higher membrane/cytoplasmic expression of SHH in ABs compared to AOTs (*p =* 0.022) and OKCs (*p* = 0.02). No significant differences were observed for the other comparisons (*p* > 0.05).

Membrane/cytoplasmic immunoexpression of SMO was detected in all cases of AB, OKC and AOT. The KW test revealed no significant differences in SMO expression between the lesions studied (*p* > 0.05) (Table 2).

At the cytoplasmic level, expression of GLI-1 was detected in all cases of AB, OKC and AOT. At the nuclear level, GLI-1 expression was found in 18 ABs (90%) and 18 OKCs (90%), while none of the AOT cases (0%) expressed this protein. The KW test demonstrated a significant difference in the median nuclear expression scores for GLI-1 between the groups of lesions (*p* < 0.0001) (Table 2). Application of the nonparametric Mann-Whitney test showed significantly higher nuclear expression of GLI-1 in ABs and OKCs than in AOTs (*p <* 0.0001) (Table 3).

Possible correlations between the expression scores for SHH, SMO and GLI-1 were analyzed in the three groups of lesions (Table 4). The nuclear and cytoplasmic expression scores for GLI-1 were positively correlated in ABs (*r* = 0.482; *p* = 0.031) and OKCs (*r* = 0.865; *p* < 0.0001) (Table 4). There was a positive and significant correlation between the membrane/cytoplasmic expression scores for SMO and the cytoplasmic expression scores for GLI-1 (*r* = 0.535; *p* = 0.015) (Table 4). In AOTs, a positive and significant correlation was observed between the cytoplasmic expression scores for GLI-1 and membrane/cytoplasmic expression scores for SMO (*r* = 0.667; *p* = 0.035) (Table 4).

**Table 2:** Sample size, median, quartiles 25 and 75, mean of the ranks, Kruskal-Wallis statistic and statistical significance (p) for SHH, SMO and GLI-1 immunolabeling scores in relation to the groups.

Location/Injury	*n*	*Median*	*Q*_*25*_ *-Q*_*75*_	*Average posts*	*KW*	*p*
*SHH*						
*Cytoplasm/Membrane*						
AB	20	3.00	2.00-3.00	32.38	9.029	0.011*
AOT	10	1.00	0.00-2.25	17.65		
OKC	20	1.50	1.00-2.00	22.55		
						
*SMO*						
*Cytoplasm/Membrane*						
AB	20	3.00	3.00-3.00	26.0	0.742	0.690
AOT	10	3.00	2.75-3.00	23.5		
OKC	20	3.00	2.00-3.00	26.0		
						
*GLI-1*						
*Cytoplasm*						
AB	20	3.00	3.00-3.00	26.33	1.313	0.519
AOT	10	3.00	3.00-3.00	27.55		
OKC	20	3.00	2.25-3.00	23.65		
						
*Nuclear*						
AB	20	2.00	1.00-3.00	27.10	22.778	<0.0001*
AOT	10	0.00	0.00-0.00	7.50		
OKC	20	3.00	2.00-3.00	32.90		

*Statistically significant (p < 0.05).

Abbreviations: AB, Ameloblastoma; AOT, Adenomatoid Odontogenic tumor; OKC, Odontogenic Keratocyst; KW: Kruskal-Wallis. Score: 0 = absence of staining, 1 = 0 to 10% of positive cells (low), 2 = 11 to 50% of positive cells (intermediate), and 3 ≥ 50% of positive cells (high).

**Table 3:** Sample size, median, quartiles 25 and 75, mean of the ranks, Mann-Whitney statistic and statistical significance (p) for SHH and GLI-1 immunolabeling scores in relation to the groups when analyzed two-by-two.

Location/Injury	*n*	*Median*	*Q*_*25*_ *-Q*_*75*_	*Average posts*	*U*	*p*
*SHH (Cytoplasm/Membrane)*						
AB	20	3.00	2.00-3.00	18.10	48.0	0.022*
AOT	10	1.00	0.00-2.25	10.30		
AB	20	3.00	2.00-3.00	24.78	114.5	0.020*
OKC	20	1.50	1.00-2.00	16.23		
OKC	20	1.50	1.00-2.00	16.83	73.5	0.248
AOT	10	1.00	0.00-2.25	12.85		
						
*GLI-1 (Nuclear)*						
AB	20	2.00	1.00-3.00	20.00	10.0	<0.0001*
AOT	10	0.00	0.00-0.00	6.50		
AB	20	2.00	1.00-3.00	17.60	142.0	0.121
OKC	20	3.00	2.00-3.00	23.40		
OKC	20	3.00	2.00-3.00	20.00	10.0	<0.0001*
AOT	10	0.00	0.00-0.00	6.50		

*Statistically significant (p < 0.05).

*Abbreviations:* AB, Ameloblastoma; AOT; Adenomatoid Odontogenic Tumor; OKC, odontogenic keratocyst; U, Mann-Whitney. Score: 0 = absence of staining, 1 = 0 to 10% of positive cells (low), 2 = 11 to 50% of positive cells (intermediate), and 3 ≥ 50% of positive cells (high).

**Table 4:** Spearman’s correlation coefficient (r) and statistical significance (p) of the immunoexpressions.

Comparison	*Ameloblastoma* *(n = 20)*		***Adenomatoide odontogenic tumor (n = 10)***		*Odontogenic keratocyst* *(n = 20)*	
	*r*	*p*	*r*	*p*	*r*	*p*
SHH (C/M) × SMO (C/M)	0.369	0.110	0.629	0.052	-0.162	0.494
SHH (C/M) × GLI-1 (C)	0.189	0.426	0.419	0.228	-0.017	0.944
SHH (C/M) × GLI-1 (N)	0.186	0.433	-	-	-0.088	0.711
GLI-1 (N) × SMO (C/M)	0.423	0.063	-	-	0.391	0.088
GLI-1 (C) × GLI-1 (N)	0.482	0.031*	-	-	0.865	<0.0001*
GLI-1 (C) × SMO (C/M)	0.327	0.160	0.667	0.035*	0.535	0.015*

*Statistically significant (p < 0.05). *Abbreviations:* C, Cytoplasm; M, Membrane; N, Nuclear.

### Discussion

The Shh signaling pathway has been extensively studied because of its known strong influence on the development of a series of embryonic tissues, including odontogenesis ^2^. Furthermore, deregulation of this pathway has been associated with the pathogenesis of different benign and malignant neoplasms ^16^ ^,^ ^17^. Analysis of Shh proteins in different cellular compartments is of fundamental importance since these proteins exert different functions depending on where they are expressed. This was the proposal of the present study. To our knowledge, this is the first study that compared the expression of SHH, SMO and GLI-1 between different cellular compartments in AB, OKC and AOT.

All studies in the literature that investigated the immunohistochemical expression of Shh proteins in different types of odontogenic lesions reported positive immunoreactivity ^5^ ^,^ ^8^ ^,^ ^15^ ^,^ ^18^ ^,^ ^19^ ^,^ ^20^. This agrees with the present findings, indicating a strong association of this pathway with the pathogenesis of the odontogenic lesions analyzed.

The staining patterns of the proteins in the lesions studied were similar to those reported in other studies. A variable staining pattern was identified in ABs. However, in some cases, the expression of SHH and SMO was more intense in peripheral epithelial odontogenic cells of the follicular and plexiform patterns, in agreement with the study of Zhang et al. ^15^. In contrast, Vered et al. ^5^ observed stronger staining in loosely arranged central cells, suggesting that these more strongly stained cells may play a progenitor role and are responsible for proliferation of the tumor. With respect to AOTs, like Zhang et al. ^15^, we found an indistinct staining pattern, with strong staining mostly in solid areas such as ductiform and rosette-like structures.

In cases of OKC, SHH was mainly expressed in cells of the basal and parabasal layers, similar to the findings of Cadavid et al. ^20^. On the other hand, SMO and GLI-1 were expressed in cells of the basal, parabasal and intermediates layers but not in the more superficial layer, suggesting that this layer does not actively participate in cell renewal. Despite the different staining patterns, the basal and suprabasal layers were intensely stained for all three proteins analyzed, which may indicate that the cells of these layers are related to the growth of this cyst. In agreement with Zhang et al. ^15^, we also suggest that the Shh signaling pathway plays a key role in the formation of the epithelial lining of this cyst.

In addition to the number of immunostained cells, we also analyzed the expression of the proteins according to cellular compartment since the function of each protein can differ depending on the site of expression. Immunoexpression of SHH was found in all AB and OKC cases and in seven AOT cases, demonstrating predominantly membrane/cytoplasmic staining. Similar results have been reported by Cadavid et al. ^20^ who observed cytoplasmic staining in syndromic and non-syndromic OKCs, and by Ge et al. ^21^ who found staining near the plasma membrane in breast cancer. Taken together, the present findings and those of the cited studies reinforce the importance of cytoplasmic and membrane localization of SHH, which triggers the activation of the pathway by binding to PTCH1, a protein present in the plasma membrane.

Also regarding SHH, significantly higher membrane/cytoplasmic expression was observed in ABs compared to AOTs and OKCs. This finding indicates that the Shh pathway might be more active in this odontogenic tumor, confirming its extremely aggressive biological behavior. This suggestion is based on the findings of Kanda et al. ^11^ who, by silencing the SHH gene, inhibited the proliferative activity of ameloblastoma cell lines. The authors suggested that the Shh signaling pathway is constitutively active in AB and exerts an antiapoptotic effect on cell proliferation in this tumor, enhancing its markedly invasive growth in adjacent tissues.

As described by Mishra et al. ^2^, Amm and McDougall ^14^ and Cadavid et al. ^20^, the Shh signaling pathway is triggered by the binding of SHH to the transmembrane receptor PTCH1, which induces the translocation of the transmembrane protein SMO to the cell surface. SMO is previously inactivated and endocytosed in vesicles by the action of PTCH1. Once activated in the membrane, SMO triggers a signaling cascade that activates GLI proteins, thus mediating the SHH signal from the cytoplasm to the nucleus of the target cell.

The immunohistochemical findings of the present study revealed both membrane and cytoplasmic immunoexpression of SMO in all cases of the three groups of lesions studied. However, there were no statistically significant differences in the expression of SMO between lesions, probably because this protein was highly expressed in all three types of lesion analyzed. These results differ from those of Zhang et al. ^15^, Vered et al. ^5^ and Cadavid et al. ^20^ who observed a low to intermediate expression pattern in cases of non-syndromic OKC. Curiously, Vered et al. ^5^ and Cadavid et al. ^20^ found high expression levels of SMO only in syndromic OKCs, suggesting that this protein might be related to the development of this lesion in nevoid basal cell carcinoma syndrome.

The present results and those reported in the cited studies indicate high membrane/cytoplasmic expression of SMO in both non-syndromic and syndromic OKCs, suggesting mutations in the SMO gene in both types of lesion. Molecular studies are necessary to evaluate mutations in this gene in non-syndromic and syndromic OKCs. Within this context, Sweeney et al. ^13^ detected mutations in the *SMO* gene in cases of AB. This discovery could help identify new molecular therapeutic targets for OKCs aimed at blocking the hedgehog pathway through *SMO*, an approach used in maxillary AB, as demonstrated by these authors.

Immunohistochemical analysis of GLI-1 showed cytoplasmic staining in most cases of the three lesions studied, while none of the AOT cases exhibited nuclear expression of the protein. Nuclear expression of GLI-1 was thus significantly higher in ABs and OKCs than in AOTs (*p <* 0.0001). It should be noted that this was the first study analyzing the expression of Shh proteins according to cellular compartment in AOTs, providing an important finding, i.e., the lack of nuclear immunoexpression, which reinforces the indolent behavior of these odontogenic tumors.

Regarding the nuclear expression of Shh proteins in ABs and OKCs, the present findings agree with the results of previous studies on these lesions ^5^ ^,^ ^11^. In addition to the cited reports, Ge et al. ^21^ studying breast cancer and Zhang et al. ^22^ studying colon cancer also demonstrated strong nuclear expression of GLI-1. These findings suggest that the nuclear expression of GLI-1 is associated with deregulation of the pathway since the protein acts as a transcription factor of target genes, leading to increased proliferation and uncontrolled cell growth. Thus, the high nuclear expression of GLI-1 may be related to both the pathogenesis and the aggressive potential of AB and OKC.

Analysis of possible correlations between the immunoexpression scores for SHH, SMO and GLI-1 in the three groups of lesions revealed a significant positive correlation between cytoplasmic and nuclear GLI-1 expression in ABs and OKCs. This finding reveals overexpression of this protein and a consequently greater regulation of the Shh pathway in these lesions, with increased nuclear expression of GLI-1 indicating marked proliferative activity of the cell.

A significant positive correlation was also observed between the membrane/cytoplasmic expression of SMO and exclusive cytoplasmic expression of GLI-1 in AOTs and OKCs. This result regarding AOTs may suggest a minor role of GLI-1 in stimulating cell proliferation in this tumor since the protein is not increased at the nuclear level, confirming the low growth potential of this lesion. However, in the case of OKCs, it is believed that the sum of this cytoplasmic SMO/GLI-1 correlation along with the strong cytoplasmic and nuclear expression of GLI-1 may contribute to the high growth potential of this cyst.

Immunohistochemistry is widely used in contemporary pathology as a diagnostic and, increasingly, as a prognostic and predictive tool. One of the major limitations of immunohistochemistry is related to the fact that the results are usually obtained by visual qualitative or semiquantitative evaluation. While this is sufficient for diagnostic purposes, measurement of prognostic and predictive biomarkers requires better accuracy and reproducibility. Further studies are required to better understand the value of immunohistochemical markers analyzed in the present study. Understanding the molecular mechanisms underlying the development and progression of the analyzed lesions may be the basis for the discovery of novel biomarkers and development of improved therapeutic strategies.

## Conclusion

In conclusion, the results of this study provide additional evidence supporting the highly proliferative behavior of AB indicated by the high membrane/cytoplasmic expression of SHH. Additionally, the high nuclear expression of GLI-1 in OKCs and ABs and its low expression in AOTs could be associated with the more aggressive biological behavior of the former compared to AOT. The results suggest that the strong expression of the Shh proteins analyzed in this study (SHH, SMO, and GLI-1) may plays a role in the pathogenesis of the odontogenic lesions studied (OKC, AB, and AOT).
